# Dr Google and the Consumer: A Qualitative Study Exploring the Navigational Needs and Online Health Information-Seeking Behaviors of Consumers With Chronic Health Conditions

**DOI:** 10.2196/jmir.3706

**Published:** 2014-12-02

**Authors:** Kenneth Lee, Kreshnik Hoti, Jeffery David Hughes, Lynne Emmerton

**Affiliations:** ^1^School of PharmacyCurtin UniversityPerthAustralia

**Keywords:** online health information seeking, health information search, health seeking behavior, consumer health information, information needs, Internet, chronic disease, patients, qualitative research, interview

## Abstract

**Background:**

The abundance of health information available online provides consumers with greater access to information pertinent to the management of health conditions. This is particularly important given an increasing drive for consumer-focused health care models globally, especially in the management of chronic health conditions, and in recognition of challenges faced by lay consumers with finding, understanding, and acting on health information sourced online. There is a paucity of literature exploring the navigational needs of consumers with regards to accessing online health information. Further, existing interventions appear to be didactic in nature, and it is unclear whether such interventions appeal to consumers’ needs.

**Objective:**

Our goal was to explore the navigational needs of consumers with chronic health conditions in finding online health information within the broader context of consumers’ online health information-seeking behaviors. Potential barriers to online navigation were also identified.

**Methods:**

Semistructured interviews were conducted with adult consumers who reported using the Internet for health information and had at least one chronic health condition. Participants were recruited from nine metropolitan community pharmacies within Western Australia, as well as through various media channels. Interviews were audio-recorded, transcribed verbatim, and then imported into QSR NVivo 10. Two established approaches to thematic analysis were adopted. First, a data-driven approach was used to minimize potential bias in analysis and improve construct and criterion validity. A theory-driven approach was subsequently used to confirm themes identified by the former approach and to ensure identified themes were relevant to the objectives. Two levels of analysis were conducted for both data-driven and theory-driven approaches: manifest-level analysis, whereby face-value themes were identified, and latent-level analysis, whereby underlying concepts were identified.

**Results:**

We conducted 17 interviews, with data saturation achieved by the 14th interview. While we identified a broad range of online health information-seeking behaviors, most related to information discussed during consumer-health professional consultations such as looking for information about medication side effects. The barriers we identified included intrinsic barriers, such as limited eHealth literacy, and extrinsic barriers, such as the inconsistency of information between different online sources. The navigational needs of our participants were extrinsic in nature and included health professionals directing consumers to appropriate online resources and better filtering of online health information. Our participants’ online health information-seeking behaviors, reported barriers, and navigational needs were underpinned by the themes of trust, patient activation, and relevance.

**Conclusions:**

This study suggests that existing interventions aimed to assist consumers with navigating online health information may not be what consumers want or perceive they need. eHealth literacy and patient activation appear to be prevalent concepts in the context of consumers’ online health information-seeking behaviors. Furthermore, the role for health professionals in guiding consumers to quality online health information is highlighted.

## Introduction

The Internet is a popular source of health information among consumers [1-4]. The vastness of health information available online affords consumers unprecedented access to health information. Such access to health information is paramount, given the global push for consumer-focused health care, particularly in the management of chronic health conditions [5-11]. Despite the pervasiveness of the Internet, several of its design features have been identified as potential impediments to consumers’ functional access to online health information [12]. As such, navigation of online health information can be problematic. The volume of health information available online can result in information overload [12-14]. The use of overly technical language to convey information, along with the volume of irrelevant content returned from search engine results, the confusing layout of many Web pages, the lack of universal quality requirements for publishing online content, and the abundance of inaccurate or misleading information, are other reported challenges [12]. Despite recognition in the literature of potential navigational issues, to date, little is known about what consumers want in order to better navigate the Internet to access health information.

A comprehensive literature review was conducted to explore interventions aimed at assisting consumers with finding quality online health information related to chronic health conditions [15]. In addition to highlighting the paucity of research in this field, a didactic approach was common among the identified interventions. In these interventions, participants were *taught* how to use the Internet, evaluate the reliability of online health information, or how to use health information databases. Such interventions appear to have been created without research into the navigational needs of consumers.

In order to understand the consumer perspective, we propose the need to explore consumers’ online health information-seeking behaviors (HISB). In order to understand consumers’ needs for finding online health information, it is essential to know what types of health information are sought online, as well as why, when, where, and how, and the actions that are taken. To date, much of the literature on consumers’ online HISB appears to examine only aspects of the concept such as exploring the types of [4,16] and reasons why [16,17] health information is sought, characterizing demographic characteristics of online health information seekers [3,4,18,19], and determining predictors of online health information seeking [1,20]. Furthermore, many studies appear to examine the HISB and online HISB of consumers from populations with particular health conditions [21], such as cancer [17,22-26], spinal cord injury [27,28], and human immunodeficiency virus (HIV) [29-31]. Studies exploring the general online HISB of consumers [3,4,16] included consumers who may not have chronic health conditions. There is thus potential to determine the online HISB and needs of consumers with at least one chronic health condition, representing a significant portion of the adult population.

Studies have suggested the interaction between consumers and health professionals to be a motivator for people to seek health information online [20,21,32-34]. Additionally, consumers’ reasons for seeking health information online include enhanced feeling of empowerment [21], perception of improved engagement with their health professionals [21,34], lack of information provided by health professionals [21,35], and dissatisfaction with consumer-health professional interactions [20]. Despite the apparent popularity of the Internet as a source of health information, literature suggests that consumers generally still value the advice given by health professionals above that of health information sourced online [2,3,21,33,36,37]. This suggests that health professionals can play an important role in consumers’ online HISB, and we anticipate that consumers’ relationships with their health professionals will be pertinent to this investigation.

Further to understanding consumers’ navigational needs, a comprehensive understanding of potential barriers to navigation is needed. Recognizing that many consumers have limited ability to find, evaluate, and use health information effectively [38-42], the concept of health literacy is relevant to this investigation. Further to this is consideration of the online environment, with consumers requiring computer and media literacies and skills to overcome the barriers to finding information online, collectively known as eHealth literacy [43]. A more comprehensive picture of the breadth of barriers impeding effective navigation of online health information can thus supplement understanding of navigational needs.

Therefore, this study aims to explore the navigational needs of consumers when searching for health information online for the purpose of self-management of chronic health conditions. To gain a more comprehensive understanding of consumers’ needs, this will be explored in the broader context of consumers’ online HISB, and potential barriers to navigation will be identified. This approach aims to provide understanding of the navigational needs of health consumers, to inform initiatives and interventions that recognize consumers’ needs.

## Methods

### Procedures and Participants

The apparent need to explore and understand the consumer perspective was approached using qualitative inquiry. Semistructured face-to-face interviews were conducted individually with health consumers. Ethical approval was granted by the Curtin University Human Research Ethics Committee (HR06/2013) and provided for each participant to be offered an AUD $25 gift card to compensate for their travel and time.

Purposive recruitment took place in August-September 2013. Participants were at least 18 years of age, conversant in English, had at least one chronic health condition, and used (or had used) the Internet to find health information related to their condition(s). While an appropriate sample size for qualitative studies should be guided by data saturation, we nominated an initial target of 20 participants, based on commonly cited sample sizes in qualitative studies [44]. The sample was partly sourced by approaching members of the public at nine community pharmacies, with permission of the respective pharmacy store managers and owners, within a practical traveling distance (25 km radius) of the research center in Perth, Western Australia. This coverage was considered to provide a broad demographic of health consumers. This study was also advertised through the student portal on the university website, a local community radio station, and two social media platforms via the university’s account.

### Interviews

Broad interview questions (Multimedia Appendix 1) surrounding the topics of online HISB and barriers and navigational needs related to finding quality online health information, were devised with reference to the literature and agreed upon by all authors to address the study objectives. Points of interest or points requiring clarification were explored using follow-up questions devised by the interviewer.

Interviews were conducted by the first author and lasted 15-50 minutes. Each interview was audio-recorded with two digital recorders and transcribed verbatim in Microsoft Word as soon as practical after each interview, supplemented by field notes. Grammatical corrections were made to improve flow and readability of the transcripts.

### Analysis

#### Overview

Transcripts were imported into QSR NVivo 10 to facilitate coding and thematic analysis. For analytical rigor, two established approaches to thematic analysis were used. First, we used a data-driven approach (Stage 1 analysis), whereby codes were generated inductively from the data [45,46]. This approach aims to minimize bias in the identification of codes and themes. The codes and themes for this approach were identified by the primary researcher. A subset of transcripts was reviewed by another member of the research team for agreement of codes and themes. On completion of the data-driven analysis, a theory-driven approach was subsequently used by the primary researcher (Stage 2 analysis). This approach required the researchers to formulate a theory about the anticipated results and then develop a provisional list or framework of codes based on the anticipated results [45,46]. Transcripts were then coded using these provisional codes, and additional codes were added if necessary. While this latter approach ensures that the codes and themes identified from the results are focused on the aims of our study, it can restrict the breadth of the findings. Hence, our purpose for using this approach is to confirm the themes identified by the data-driven approach.

Each approach to thematic analysis was further divided into manifest and latent levels of analysis. These two levels reflect how themes are identified. As themes can be broadly defined as patterns found in information, it can range from patterns that are directly observable in the information such as statements made by participants (manifest level), to patterns underlying the information such as potential meaning behind why participants made certain statements (latent level) [45]. We undertook the following specific processes.

#### Stage 1 Analysis: Data-Driven, Manifest Level

While reading the first interview transcript, codes were identified and created. Codes created from this transcript formed the skeleton of our coding framework, which was continuously revised as more transcripts were coded. Once all transcripts were coded, the codes were grouped into broader categories for identification of themes.

#### Stage 1 Analysis: Data-Driven, Latent Level

On completion of the manifest-level analysis, the latent-level analysis was conducted. Here, the manifest-level themes identified were continuously compared against all the interview transcripts in attempts to identify underlying patterns (latent themes).

#### Stage 2 Analyses: Theory-Driven, Manifest and Latent Levels

**Figure 1:**
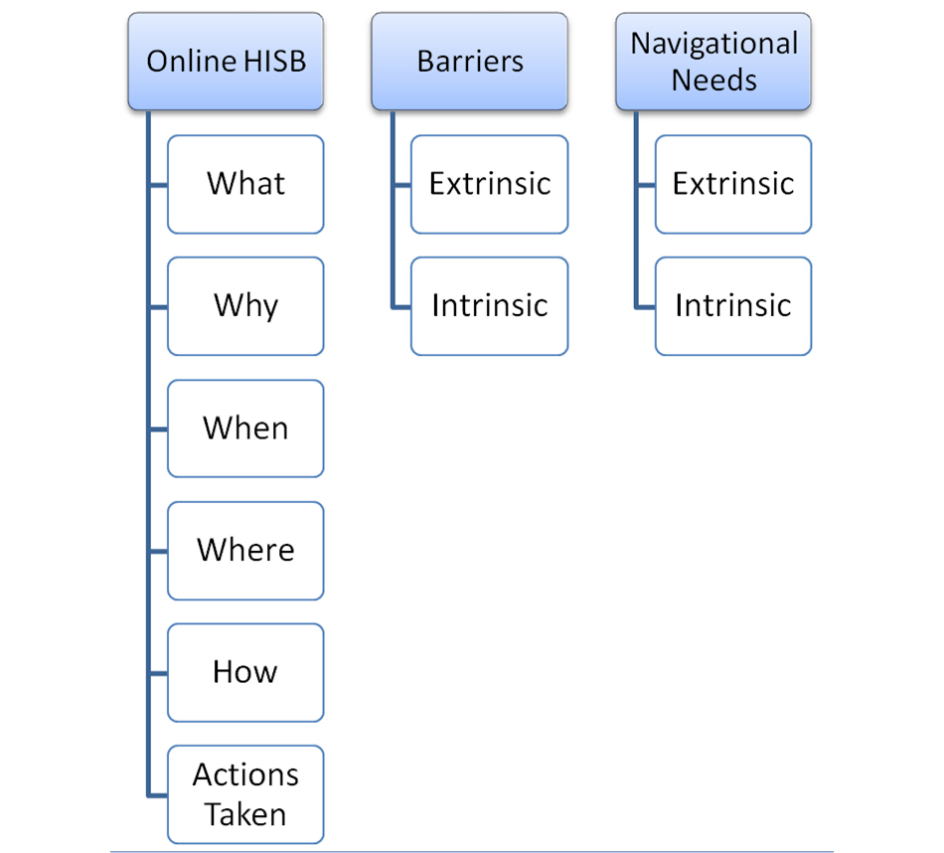
Framework for thematic analysis using a theory-driven approach.

Figure 1 illustrates the theory that we devised based on our anticipated results. As codes had already been created in Stage 1, a separate QSR NVivo 10 project was established so new codes could be created for this stage. As illustrated in Figure 1, our theory-driven approach categorized online HISB according to *what* types of health information are sought online, *why* health information is sought online, *when* health information is sought online (eg, prior to or after consultation with a health professional), *where* consumers go to obtain online health information (ie, their source(s) of information), *how* consumers go about obtaining online health information (eg, their search strategies), and *actions taken*, that is, what consumers do with the information they find online. We anticipated that barriers to finding online health information could be broadly coded as either extrinsic or intrinsic. For this study, we defined “extrinsic” barriers as those related to the environment or health system. This includes health professionals, health policies, and the design of the Internet/Web pages. We defined “intrinsic” barriers as those pertaining to individual health consumers, such as their motivations to seek health information, and their knowledge medical conditions and their management. These definitions were based on a recent report [47] on health literacy whereby the definition of health literacy was divided into “individual” and “environmental” components. We believe that these two components would apply to our study. We also anticipated that participants’ navigational needs would be extrinsic and intrinsic in nature. These categories formed the framework under which subsequent codes were categorized.

### Data Saturation

Data saturation was defined by the researchers of this study as the point whereby no new codes could be generated from the data-driven approach. In practice, this point was determined as the participant transcript after which no new codes, relevant to the study, were created in QSR NVivo 10.

## Results

### Summary

Of the consumers recruited from the nine community pharmacies, 20 initially agreed to participate, of whom 11 attended the interview. Of the remainder, 7 consumers could not be reached, 1 consumer asked to postpone the interview but later retracted due to ailing health, and another did not attend the scheduled interview and could not be contacted thereafter.

After learning of the study via the aforementioned media channels (student portal on the university website, a local community radio station, and two social media platforms via the university’s account), 13 consumers enquired about participation. Of these, 8 people agreed to participate, but 1 did not attend the scheduled interview and could not be reached thereafter.

In total, 17 eligible participants completed the interview: 10 participants recruited from pharmacies, 4 via community radio, and 3 via university advertising channels. An additional recruit was excluded due to ineligibility identified during the interview. There were 9 female and 8 male participants. The age of participants ranged from 19-85 years, with the most common age category being 50-60 years. The majority of participants appeared to be native English speakers. Participants comprised 3 university students, 8 in the workforce, and 6 retirees. The participants’ diagnosed medical conditions were not formally recorded for privacy reasons; however, their conditions revealed during the interviews were broad ranging, and a majority of participants reported having more than one chronic health condition.

Data saturation was perceived by the 14th interview; no new codes relevant to this study were generated from the remaining three interview transcripts.

### Manifest-Level Analysis

Tables 1 and 2 reveal a wide range of responses related to *what* types of online health information were sought, *why*, *when*, *where,* and *how* online information was sought, and what consumers reported doing with the health information they found online (*actions taken*).

As indicated in Table 1, participants most commonly reported seeking health information related to understanding their medical conditions and the medications prescribed by their health professionals (*what*). Common reasons for seeking information online were to be more informed about their health and were commonly related to consultations with their health professionals (*why*). The most common time in which participants were more likely to seek information (*when*) was after consulting a health professional.

As indicated in Table 2, despite typically searching for health information after consulting a health professional, the most common approach was the use of a search engine (*where* and *how*). Once participants obtained health information, their actions appeared to largely revolve around the consultations with their health professionals in the sense that participants either decided to relay their findings to their health professionals or make decisions about whether to consult their health professionals for advice or medical attention (*actions taken*). Some participants also reported that the information sourced online assisted in making decisions about whether to use therapeutic products or to trial lifestyle modifications to supplement advice given by their health professionals.

**Table 1:** Online HISB: what, why, when (responses are listed in approximate order of frequency from most to least frequently mentioned).

What	Why	When
Medicines or medical devices—includes side effects, and indications for the products	To be more informed	Following a consultation with a health professional
Medical conditions	To clarify/verify information discussed during consultation	Before a consultation with a health professional
Lifestyle information (eg, information on diets and exercise)	Because the Internet is accessible	When required
Information about individual health professionals, medical clinics and hospitals (eg, appropriate specialists for particular conditions)	Emotional support, eg, to read about experiences of others with the same condition(s)	Before and after a consultation
Natural products	Out of interest	
Information about disease-specific associations, eg, Cancer Council and Diabetes Australia	Disagreement with points made by a health professional	
Medical glossary	To seek alternative/additional treatment options	
	Insufficient information provided during a consultation	
	Urgency to know	
	To take charge of one’s life	
	Self-management of a perceived minor condition, eg, common cold	
	Limited time during consultation	
	To have written information to read	
	Infrequency of interaction with health professional	

**Table 2:** Online HISB: where, how, actions taken (responses are listed in approximate order of frequency from most to least frequently mentioned).

Where	How	Actions taken
Search engines (eg, Google, Bing)	Start with results from a search engine	Discuss information with a health professional
Disease-specific association websites	Direct URL	Decide whether to consult with a health professional
Forums/support groups	Start with websites recommended by others	Decide whether to purchase/use a medication or natural product
Wikipedia		Trial lifestyle modifications
General health websites (eg, Better Health Channel)		
Website recommended by health professionals		
Research databases		
eNewsletters		
Private health insurer websites (eg, Medibank)		

Table 3 summarizes participants’ self-reported barriers to online HISB. Table 4 summarizes participants’ reported navigational needs for finding online health information.

Participants’ self-reported barriers to online HISB (Table 3) were able to be categorized into “extrinsic” and “intrinsic” subcategories. This suggests that our findings are aligned with our theorized framework illustrated in Figure 1. Extrinsic barriers included issues with the information or presentation of information online, the volume of information available online, and issues with the relationship between consumers and their health professionals. Regarding intrinsic barriers, limited “eHealth literacy skills” was the prevalent theme.

When participants were asked about their navigational needs (Table 4), their comments focused on extrinsic needs and encompassed improving access to, or transparency of, quality information, reducing/blocking access to poor quality information, and involving health professionals in guiding consumers to quality health information. No intrinsic navigational needs were reported by our participants.

**Table 3:** Participants’ self-reported barriers to online HISB.

Category	Feedback
Extrinsic	Availability/accessibility of content, eg, difficulty in accessing content published in research journals
	Use of medical jargon
	Inconsistency of information across different sources
	Volume of information available
	Poor interaction/relationship with health professional, eg, low level of trust in the advice given by health professionals
Intrinsic	Limited eHealth literacy skills
	Limited knowledge of credible websites
	Unsure of information need
	Limited time available to search for information
	Lack of motivation to search for information

**Table 4:** Participants’ self-reported navigational needs.

Category	Feedback
Extrinsic	Greater availability/accessibility of content
	Single/designated destination for credible information
	Health professionals’ input, such as providing an annotated list of potentially useful websites
	Blocking untrustworthy websites
	Stricter rules for publishing information online
	Improved webpage layout and features
Intrinsic	None reported by participants

### Latent-Level Analysis

Our latent-level analysis revealed that there were three themes underlying one or more aspects of our participants’ online HISB, and barriers and navigational needs for finding online health information: trust, patient activation, and relevance. In particular, these three themes appear to provide potential reasons as to why participants chose certain sources of information and why they were motivated to search for health information online, and they reinforce the important role online health information plays in the self-management of chronic health conditions.

### Trust

Throughout the interviews, the idea of trust became evident as a factor associated with online HISB. This idea of trust encompassed both trust in health professionals, and trust in certain online sources such as search engines. Trust in advice given by health professionals is exemplified in the following quotations:

If it’s something that the doctor has prescribed for me, generally I don’t research that, because I think the doctor has decided that “that’s what I want”, and he decided what the dose is and told me what the side effects might be—so I don’t actually go into that.

P7

The Internet is part of my life medically, but only a relatively small part. I rely mostly on my GP and specialist for most things.

P12

Yes, and [my doctor is] very good with latest research and things. He will say, “Oh I read this, and that doesn’t work anymore”. What was that thing that I was going to talk to him about next time that’s been recently in the TV—is it Statins for the high blood pressure? That’s one thing I will ask him. So I’d rather trust him than say, 60 Minutes [television show].

P17

Despite this trust in their health professionals, participants still searched for health information online. This suggests that the idea of trust also applied to online sources. From one participant reflecting on Wikipedia:

Generally, I’d say I trust it, but I know it can be very variable because it’s written by all sorts of people. They do have a team of writers, but they are, as the name implies, almost anyone can add to it. So you’ve got to look carefully at the qualifications of who’s written that particular piece.

P7

Further to the topic of trust in online sources, search engines such as Google were mentioned by many participants as a trusted source. The following two examples suggest that the participants trust the Google search engine to provide them with a starting point to finding relevant information.

When asked how online information is sought:

For example, for glandular fever, I will put in “recurring glandular fever”. When you do it in Google, it virtually thinks ahead for you. So it’s fantastic in that way because maybe you haven’t thought of exactly the right words and it comes up with half a dozen—it triggers off this set of words and then you just click on the one that’s closest to the one that you’re thinking.

P11

When asked how online information is sought:

In general terms, I start off with Google and see where that takes me.

P13

However, while participants generally appeared to trust certain online sources of health information, the majority of participants still sought clarification with health professionals for their discipline expertise. The following participant mentioned seeking clarification with their doctor after obtaining health information online:

I had an issue last year where my feet were swelling and I was getting a bit of a pigmented pattern. I looked that up to see what it was…I thought “Oh, should I be worried? What’s this? I should go mention it to my doctor”…It prompted me to say “I better go and ask a question about it next time I see him [the doctor]”.

P4

A lack of trust appears to contribute to some of the identified barriers in Table 3. For example, individuals may not trust online health information because they are unable to appraise the information for its reliability. This could be due to an individual’s limited eHealth literacy, among other factors.

When asked for his/her opinion about health information found online:

I don’t put a great deal of trust into it because, you know, so many different things come on the Internet. How do you weigh the worth against another when it’s not your field?

P8

Regarding navigational needs, the theme of trust appeared to be a factor influencing participants’ comments. For example, one participant commented that their doctor provided an annotated list of relevant websites, which the participant found valuable:

He actually gave me a list of websites that are good ones to use...I like the fact that I was given a choice, given a list, so I could go through them all and choose one for me. I wasn’t just given one and told “this will suit all your needs”—because it’s never really going to and I guess in that sense, I did go through and decide to pick the one that I liked. But you know, you could pick and choose and compare.

P15

While many participants reported that they trust a variety of online information sources, most of these participants also reported that they trust one or more of their health professionals. This theme of trust appeared to underlie participants’ online HISB and barriers and navigational needs for finding online health information. Interestingly, it appears that trust in health professionals prevailed over trust in online sources.

### Patient Activation

Patients or health consumers are considered to be activated if they “believe patients have important roles to play in self-managing care, collaborating with providers, and maintaining their health. They know how to manage their condition and maintain functioning and prevent health declines; and they have the skills and behavioral repertoire to manage their condition, collaborate with their health providers, maintain their health functioning, and access appropriate and high-quality care” [48].

The theme of “patient activation” aligned primarily with the reasons for consumers to seek online health information: an urgency to know, to be more informed, to find alternative treatment options, to take charge of one’s life, and to manage self-perceived minor conditions. Components of this definition were reflected in comments from participants.

When asked why the participant sought information online:

Just to look for information that my doctor wasn’t able to tell me…They didn’t know what the cause was. So I was really adamant to find out what the possible cause would be.

P1

When asked about whether the participant experiences difficulty finding health information online:

I generally find that I can find what I want pretty quickly. As I say, there’s all sorts of things with Google. It’s quite massive the amount of information that is there, but you’ve got to use a little bit of intelligence to get through it.

P2

On the topic of why health information is sought online as opposed to seeking a health professional:

There’s sometimes a waitlist…if I have to wait a week to see a doctor, I lose interest, so I kind of go, “ok, I’m going to research this”, do it and that’s it.

P3

When asked why the participant sought information online:

More so clarification and to get information…because I want to be informed… I don’t want to be a passive patient…I want to know what’s going on, I want to make informed decisions. So if they decide on a particular course of treatment, I want to know what’s going on and if there is an alternative.

P16

In terms of patient activation influencing barriers to finding online health information, one participant claimed:

I’m not terribly patient of the Internet. I would turn it off and ask someone. I think some people will sit there because they’re happy looking at a screen and clicking forever, whereas if I can’t get something reasonably quickly, or know where to start, I’ll just get a book off the shelf or see someone.

P6

This appears to reflect the participant’s lack of motivation to use the Internet to find health information. However, it is important to note that this participant still appeared to demonstrate a degree of activation from being motivated to seek health information from other sources.

While participants highlighted their desire for health professionals to play a greater role in assisting their navigation of online health information (Table 4), many participants also expressed the need to improve various aspects of the Internet, such as blocking untrustworthy websites. This reinforces the desire for participants to use the Internet to find health information and suggests patient activation is a potential factor having an impact on their navigational needs.

### Relevance

While some participants reported that they also looked online for information about health topics that were of interest but were unrelated to their health issues, the primary reason for searching online was to find health information relevant to their condition(s). This suggests that our participants appeared to value the role online health information can play in self-management of chronic health conditions.

When asked what types of health information is sought:

Well, usually it was for pain patches, because I want to investigate about putting on weight with them, because I have a lot of fluid retention…So I want to look at that and see.

P5

When asked what the participant would do regarding an information need:

I would look at the Internet a number of times, because there’s so much conflicting information out there. And then I would try and keep narrowing it down to things that may be helpful for my condition.

P15

In terms of barriers to finding online health information, the volume of irrelevant information can impede access to relevant online health information. One participant commented that this can be particularly important when one has comorbidities:

There’s so much information out there. It’s having more than one medical condition, for me, makes it complicating. For example, I’ll be looking up depression and diabetes, and there’s not always a cross-match to compare them.

P3

The navigational needs listed in Table 4 suggest participants’ desire to be more able to find relevant and reliable online health information, as illustrated by the following participant:

I’d love to have a search function that can say “I want to hear about blah, blah and blah, but not blah”.

P17

## Discussion

### Principal Findings

Our study suggests that the didactic emphasis of existing interventions is not in line with consumers’ needs when searching for online health information. Our participants’ navigational needs were extrinsic in nature. They wanted to be able to better navigate online health information by improving systems supporting their health information-seeking activities. These systems include design features of the Internet that have been reported [12] as potential impediments to functional access, as well as health professionals guiding consumers to appropriate online resources. This finding reinforces the need for greater efforts into addressing such design issues of the Internet, as well as suggesting that health professionals can play a role in consumers’ navigation of online health information.

Regarding the online HISB of consumers with chronic health conditions, we report that such behaviors commonly revolve around (either before, or more commonly after) consultations with health professionals. Various reported reasons *why* our participants sought information (Table 1) and *actions taken* (Table 2) by our participants appear to be in line with a study of consumers’ online HISB in the context of medical consultations [21,34]. Given the long-term management requirements for chronic health conditions and the need for regular appointments with health professionals, it appears reasonable that online HISB is aligned with health professional consultations. This finding reinforces the importance of health professionals in influencing the online HISB of consumers with chronic health conditions and further supports literature reporting consumers’ trust in health professionals with regards to health information [2,3,33,36,37].

Regarding *how* online health information is found (Table 2), search engines were often the first port of call. This finding confirms other studies whereby search engines have been identified as a dominant source of health information [16,49-51]. Our findings suggest that this could be attributed to identified intrinsic barriers such as a lack of awareness of relevant and reliable websites. However, further studies are recommended to determine other potential reasons. Given the prevalent use of search engines to acquire health information and some participants’ expressed “trust in Google” to provide relevant results, it appears that search engines can also play an important role in influencing consumers’ online HISB. This is reinforced by the themes of trust and relevance, which we identified as two of the three themes underlying consumers’ online HISB and navigational needs.

In terms of the extrinsic barriers (Table 3), our findings suggest that greater involvement by health professionals could assist in minimizing such barriers. For instance, by providing consumers with greater guidance on where to go for quality resources that are pertinent and comprehensible to the individual consumer, the issues of medical jargon, volume of information, and inconsistency of information across different sources may be addressed. In doing so, this could also perceivably improve the health professional–consumer relationship. Such involvement is consistent with one or more of participants’ navigational needs (Table 4). Similarly, given the prominent use of search engines in the context of online HISB and consumers’ navigational needs, improving filtering of poor quality websites by search engines could help address one or more of the identified barriers (Table 3) and is also in line with participants’ navigational needs (Table 4). These suggestions require further investigation to ensure validity in our claims.

Our study identified eHealth literacy skills as a prominent intrinsic barrier to finding online health information. This prevalence is suggestive of the need to address issues of Health literacy and eHealth literacy, as suggested by the multitude of studies conducted in these fields [38-41,43]. We also identified that the theme of patient activation underlies consumers’ online HISB. Given the prominence of eHealth literacy as a barrier, as well as the importance of patient activation in consumers’ online HISB, there may be associations between consumers’ online HISB, eHealth literacy and patient activation, and their role in self-management of chronic health conditions. Further investigation is required to explore the possibility of such associations.

The themes of trust, patient activation, and relevance, identified via latent-level analysis, underpinned one or more aspects of our participants’ online HISB and barriers and navigational needs for finding online health information. While the theme of trust has been identified in various studies as a predictor of online HISB [1,20,32,52], the concept of patient activation appears less studied in the online health information context. We propose that patient activation will remain crucial in light of social trends towards self-care. One would expect that consumers with chronic conditions would most commonly search for information relating to their own conditions. However, it is noteworthy when considering the plethora of online information and potential challenges for consumers in identifying relevance of information. While further studies are required to examine the significance and interrelationships of these three themes, we have provided a framework for future initiatives to support consumers in their navigation of online health information.

### Strengths and Limitations

A key strength of this study lies in our use of two approaches to analysis (data-driven and theory-driven analyses) to assist with consolidation of the themes. Further to minimizing bias in analysis, as mentioned above, the use of a data-driven approach has been suggested to offer greater criterion and construct validity over a theory-driven approach [45]. A limitation of the data-driven approach is potential identification of codes and themes that may not be relevant to the study objectives [45]. We believe that combining these two approaches improves the rigor of our analysis by mitigating limitations from each individual approach. As such, we believe our findings can be considered more robust compared to using each analytical approach separately.

By separating manifest and latent-level themes, and by demonstrating what we classified as manifest and latent-level themes, we have added transparency to our methods of analysis. This facilitates peer critique of our interpretation and assessment of the robustness of our findings.

While many studies focused on HISB and online HISB in the context of specific chronic conditions [17,22-31,53-55], our study qualitatively explored online HISB for a variety of chronic health conditions. Despite the broader focus of our study, manifest level themes identified (Table 1) including the types of and reasons why online health information is sought, is in line with findings from various disease-specific studies. This corroboration of findings, despite differing research designs, settings, and sampling approaches, suggests consistency of our interpretation of our results and robustness in our findings. Our study therefore provides a foundation to better understanding the overall concept of online HISB.

At the time of writing, we had not identified any other study that attempted to identify consumers’ navigational needs when searching for online health information. Hence this study provides the foundations for investigation into the navigational needs of health consumers in the context of online health information seeking.

While various means of recruiting participants were used, all participants were from the metropolitan area in Perth, Western Australia. This limits the generalizability of our findings. Further, the nature of qualitative research means that generalization of findings is not appropriate. Nevertheless, the diversity of our participants’ backgrounds, the number and types of chronic health conditions of our participants, and the match between our prominent manifest level themes with other studies suggest that our findings could be transferable to a wider population of consumers with chronic health conditions who use the Internet to find health information. Quantitative studies may be a useful supplement to our qualitative data.

A further limitation to our study is that our qualitative approach does not allow statistical analysis of interrelationships between manifest-level themes. Our data suggest potential relationships between various manifest-level themes and latent-level themes. A quantitative study is recommended for confirmation.

### Further Research

Our research uniquely explores consumers’ perspectives on how they would like to better navigate the Internet to find online health information, for the purpose of self-management of chronic health conditions. A comprehensive literature review highlighted that previous studies focused on delivering educational-type interventions [15]; however, our study reveals that consumers may prefer other avenues. Given that this, to our knowledge, is the first study inviting the consumer perspective, we recommend further investigation into consumers’ navigational needs for online health information, to inform practical solutions.

Further, the prominence of the concepts of online HISB, eHealth literacy, and patient activation in our study warrants further exploration. Other studies have examined the issue of eHealth literacy alone [43,56-58], and one study examined the interplay between health literacy and patient activation [59]. A more comprehensive understanding of these three concepts could further assist in addressing the navigational needs of consumers.

Due to the qualitative data, statistically significant correlations between our identified themes cannot be examined. Quantitative research is in progress by the researchers of this study to examine potential correlations between the prominent themes identified in this study.

The navigational needs for finding online health information, as recommended by participants, appear to address only the extrinsic barriers identified in this study. Future research could investigate reasons for this finding.

### Conclusions

This study explores consumers’ navigational needs for finding online health information and identifies various barriers to consumers’ online HISB. In doing so, it highlights that consumers may desire interventions other than the educational-type interventions provided to date and highlights avenues for future research. eHealth literacy and patient activation were prominent themes in the context of online HISB and are possibly related.

Of particular note, our findings suggest consumers’ desire for health professionals to play a role in guiding them to find relevant and reliable online health information. Consumers will continue to source health information online, and those motivated to source such information require assurance of its accuracy. By providing an understanding of how consumers can potentially be assisted in finding quality online health information, our study serves as a foundation towards assisting consumers with better self-management of their chronic health conditions.

## Multimedia Appendix 1

Interview guide.

## Abbreviations

HISB: health information-seeking behaviors
HIV: human immunodeficiency virus
