# Supplementary material for: Dr Google and the Consumer: A Qualitative Study Exploring the Navigational Needs and Online Health Information-Seeking Behaviors of Consumers With Chronic Health Conditions
Source: J Med Internet Res. 2014 Dec 2;16(12):e262. doi: 10.2196/jmir.3706 (PMC4275480; doi:10.2196/jmir.3706)
Supplement: Supplementary file 1 [file jmir_v16i12e262_app1.pdf]

INTERVIEW GUIDE

Hi [insert name of participant].

This interview is being conducted so that I can learn more about how you find health information online, and any problems you experience. If it is okay with you, I will be recording our conversation using an electronic voice recorder. The purpose of this is so that I can get all the details, but at the same time, be able to pay full attention to you during this interview. I assure you that all your comments will remain confidential. I will be compiling a report which will contain comments from all the interviews I've conducted, but there will be no references to an individual. If you agree to this interview and the voice recording, please sign this consent form...

1) What kinds of health information do you look for when you go on the Internet?
   a. PROMPT: e.g. information about your condition(s), medication information, health services available etc?
   b. Example follow-up question: do you look for any other kinds of health information online? If so, what are they?

2) When do you go online to find health information?
   a. Example follow-up question: Do you normally find what you are looking for? Why/why not?

3) How do you find the health information that you are looking for on the Internet?
   a. PROMPT: e.g. do you use search engines, ask a healthcare professional for advice on good websites, or do you ask your friends?
   b. Example follow-up questions: Do you usually use the same method of finding health information on the Internet? Why/why not? Do you think that your method of finding health information on the Internet could be improved? Why/why not?

4) Do you ever have difficulties when using the Internet to find health information?
   a. PROMPT: What sort of difficulties?
   b. Example follow-up question: Do you seek help when you do experience difficulties? Why/why not?
   c. If participant respond that they seek help, ask them WHO they seek help from.

5) How satisfied do you usually feel with the health information you find on the Internet?
   a. PROMPT: ie do you feel that you've found what you're looking for?
   b. Follow-up questions: Do you wish for some sort of assistance to help you find what you want to find on the Internet? In what way? Help from a person, a computer program etc? What would make it easier for you to find health information on the Internet?

6) For someone with the same health condition as you what websites would you direct them to?
   a. Example follow-up question: what is it about those websites that makes you want to direct others to it?

NB: PROMPT questions are only asked if the participant is unable/unsure about how to answer the question(s). When giving examples to 'prompt' the participant, ensure minimal examples are given so as to minimize potential for bias of responses. Also, make it clear to the participant that the examples given are used to explain the question and are not suggestions.
